# Molecular and Supramolecular Interactions in Systems with Nitroxide-Based Radicals

**DOI:** 10.3390/ijms20194733

**Published:** 2019-09-24

**Authors:** Maria Cristina Buta, Ana Maria Toader, Bogdan Frecus, Corneliu I. Oprea, Fanica Cimpoesu, Gabriela Ionita

**Affiliations:** 1 Institute of Physical Chemistry, Splaiul Independentei 202, 060021 Bucharest, Romania; butamariacristina@gmail.com (M.C.B.); atoader@icf.ro (A.M.T.); bogdan.frecus@gmail.com (B.F.); 2 Department of Physics, Ovidius University, 900527 Constanţa, Romania; cornel.oprea@univ-ovidius.ro

**Keywords:** EPR parameters, host-guest complexes, exchange coupling, zero field splitting (ZFS), hyperfine coupling, computational chemistry, density functional theory (DFT) methods, basis sets, Gaussian and Slater type orbitals, plane wave methods

## Abstract

Nitroxide-based radicals, having the advantage of firm chemical stability, are usable as probes in the detection of nanoscale details in the chemical environment of various multi-component systems, based on subtle variations in their electron paramagnetic resonance spectra. We propose a systematic walk through the vast area of problems and inquires that are implied by the rationalization of solvent effects on the spectral parameters, by first-principle methods of structural chemistry. Our approach consists of using state-of-the-art procedures, like Density Functional Theory (DFT), on properly designed systems, kept at the border of idealization and chemical realism. Thus, we investigate the case of real solvent molecules intervening in different configurations between two radical molecules, in comparison with radicals taken in vacuum or having the solvent that is treated by surrogate models, such as polarization continuum approximation. In this work, we selected the dichloromethane as solvent and the prototype radicals abbreviated TEMPO ((2,2,6,6-Tetramethylpiperidin-1-yl) oxyl). In another branch of the work, we check the interaction of radicals with large toroidal molecules, β-cyclodextrin, and cucurbit[6]uril, modeling the interaction energy profile at encapsulation. The drawn synoptic view offers valuable rationales for understanding spectroscopy and energetics of nitroxide radicals in various environments, which are specific to soft chemistry.

## 1. Introduction

This work is dedicated to state-of-the-art computational experiments exploring the interactions that are specific to the chemistry of nitroxide-based spin probes [1,2]. We will regard this topic as belonging to the nanoscale, implying complex systems, radicals and solvents, or host-guest associations. Assemblies of medium-sized molecules are literally in the range of nanometres. In fact, we will play at the border between molecular and supramolecular regimes, keeping yet the advantage of analysis and prediction available by the nowadays electron structure methods. In the face of large and complex systems, such as molecules and solvent surroundings, proteins, or other polymers of life, the rush approach turns out to be based on simplified methods, such as molecular mechanics (MM) [3,4] and large outputs, as is the case of molecular dynamics (MD) [5,6]. 

Placing our interests in the domain of molecular magnetism [7,8,9], we will take pedantic steps checking the interaction of nitroxide-type spin carriers [10], with the solvent or host molecules, in model circumstances, treated in several computational experiments. A prototypic example is the TEMPO molecule ((2,2,6,6-tetramethylpiperidin-1-yl)-oxyl), many derivatives that are based on this moiety being produced and studied. 

The solid-state physical chemistry of such systems is very rich, showing ferromagnetic [11] or antiferromagnetic properties [12,13,14], magnetic phase transitions [15,16], all of these with mechanisms due to supramolecular effects [17,18,19,20,21,22]. The challenges are higher in the tentative account of magnetic measurements for systems in solution [23], where one might no longer benefit from the knowledge and stability of molecular and supramolecular structures, like in crystals, facing innumerable and transient possibilities of association. Our analyses are throwing some cones of light in certain aspects of such problems, although taken on idealized static systems. 

The TEMPO derivatives are relevant in biochemical respects, acting as antioxidants [24], or being used as spin probes, which are able to detect changes and variations taking place in complex environments, by analysing subtle details from the Electron Paramagnetic Resonance (EPR) measurements [25,26,27]. The experimental studies of one of us were about the supramolecular and covalent gels, such as isocyanate end-capped polyethylene glycol (PEG), the EPR measurements being the principal tool in analysing the properties of the molecular environments and the dynamic behavior of gels. The used spin labels were the TEMPO and adamantane-TEMPO radicals [28,29,30]. Additionally, we employed TEMPO units functionalized in the position 4 (opposed to NO group) by 5- and 7-doxyl-stearic acids, to monitor and interpret the thermal degradation of bovine serum albumin, speculating possible applications in protein purification technologies [31,32].

Similarly, the EPR probing was found to be valuable in the pursuit of ophthalmological treatments for keratoconus or dry eye syndrome [33], the clue being the access of the spin carriers to specific proteins. The EPR spectroscopy and the fluorescence investigations indicated that, at the molecular level, the nitroxide groups exhibit a quenching effect on the fluorescent molecules. The fluorescent systems that we investigated via these methods contain a pyrene group that is attached by polyether chains to TEMPO fragments. We also analysed their behavior in cyclodextrin assemblies, rationalizing the variation of the quenching effect with the linker length and flexibility [34].

Although we cannot address the full complexity of the phenomena implied in such measurements, we aim to chart the spin properties of interacting nitroxide-based radicals in different idealized model systems. We will call orbital paradigms, customary in the molecular magnetism of transition metal complexes, [35,36], which, to the best of our knowledge, were not fully exploited in the magnetochemistry of stable radicals [37,38]. 

In spite of the fact that many experimental studies in the chemistry and EPR spectroscopy of nitroxide systems are complemented with state-of-the-art calculations [39,40,41], usually by Density Functional Theory (DFT) [42] methods, a systematic study of orbital factors and related regularities was not yet performed. We try to fill this gap, approaching selected situations of mutual placement of spin carriers in two independent TEMPO radicals. Besides, we will consider the issue of interactions through a solvent molecule, in comparison with the through-space parameters of exchange coupling and Zero Field Splitting (ZFS) due to dipolar interactions.

The structure-property relationships of interacting nitroxide-based radicals would be relevant for various situations when considering crystalized solids of such compounds. However, for chemical purposes, we must face the complex cases where the interaction takes place in solvent, therefore implying many side-effects due to the dynamic environment. A frequent procedure in quantum chemical calculations is to account the solvent by certain approximations usually built-in the given code, such as the so-called Polarizable Continuum Model (PCM), belonging to the Self Consistent Reaction Field (SCRF) class [43]. However, these are tributary to simplified assumptions that act as surrogate for a general energy balance, but they may not work for other specific purposes, as it is our interest in the estimation of the spin Hamiltonian parameters. To the best of our knowledge, a critical and systematic discussion on this topic was not yet carried out. 

In this work, we will compare selected conformations of the radical-solvent-radical systems with the models keeping the same relative placement of the radical-radical subsystem, while considering the case of dichloromethane as solvent. Naturally, in advance of any calculation, one might suspect that the SCRF PCM and related options are not able to account the subtle details of long-range exchange coupling, since these are not providing the specific orbital interaction channels that are intervening from the side of the solvent molecule. Besides, one may expect that the exchange coupling between two radicals, communicating through an interposed solvent molecule, is strongly dependent on the rotational degrees of freedom of the solvent fragment, a fact that cannot be considered in the overall schemes approximating the solvent environments in the ab initio frames. Thus, a part of our critical analysis launches caveats regarding the yet limited availability of the first-principles approaches at the nanoscale, which cannot be covered with the common simplified methods in current use. 

In the other section we will assess the energy of encapsulation of TEMPO units inside toroidal host nano-sized molecules, cucurbiturils and cyclodextrins. The modeling of spectroscopic and structural properties of nitroxide spin-labels enclosed in various environments or solution is typically performed at the level of Density Functional Theory/Molecular Mechanics (DFT/MM) hybrid methods [44,45,46]. The interaction between the spin-label and the environment is treated by approximate electrostatics, point charges [47], or fitted multipoles [45], while the spin-label is ascribed to the quantum mechanical (QM) region and the environment forms the MM part. To provide a better description, one of the authors advanced a more complete description of the interactions between the QM and MM regions [48,49], including polarization effects that are described within the embedding response framework. Typically, previous studies rely on the so-called integrated approach [50,51], in which the dynamics of the system is simulated by molecular dynamics, while the molecular properties are obtained based on snapshots that were extracted from the MD trajectories. This approach is advantageous, because it allows for the treatment of complex systems such as encapsulation complexes in solution, but poses an inherent drawback, because the QM region is of limited extent. In this paper, we complement previous studies approaching the methodological issues of about the host-guest formation energies in different DFT computational settings. 

## 2. Results and Discussion 

### 2.1. Model Geometries of TEMPO Dimeric Assemblies. Through-space vs. Solvent-mediated Exchange Coupling

**Figure 1:**
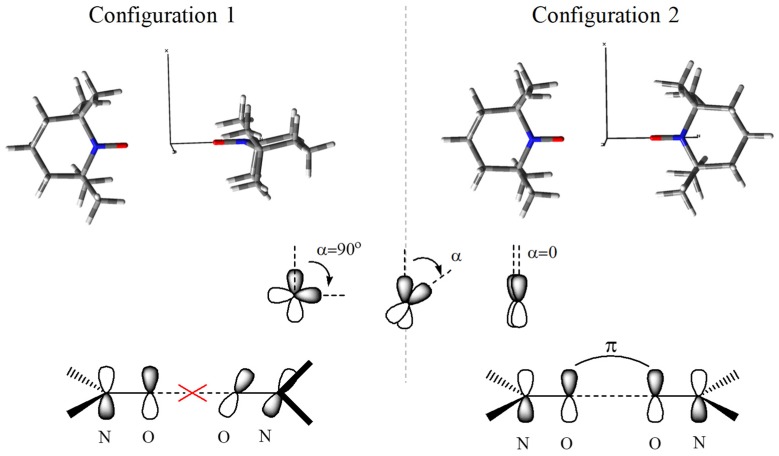
Scheme of molecular orientations and orbital overlap patterns, imposing co-linear NO groups in a TEMPO dimer, labelled “Configuration 1” (left side) and “Configuration 2” (right side). The middle inset suggests the general angle between p-type orbitals viewed along the common N-O axes. The red color is for the oxygen atom, blue for nitrogen, white for hydrogen and grey for carbon.

We start the debate choosing several model geometries for a pair of TEMPO radicals, marking the distinct inter-molecular orbital overlap encounters and their relationships with the exchange coupling. Figure 1 shows two configurations realized with TEMPO dimers, imposing the colinear placement of the two NO bond lines. The upper part of the figure depicts the realistic molecular geometries (given at the *r*_OO_ = 6 Å separation between the oxygen atoms), with the bottom line suggesting the implied types of orbital overlap. The scheme that is inserted in the middle of Figure 1 illustrates the angle between the lobes of the p-type components in the two moieties (on the oxygen atoms). The right side with parallel alignment is denoted by the α = 0 angle, the left side corresponding to the orthogonality at α = 90°.

**Figure 2:**
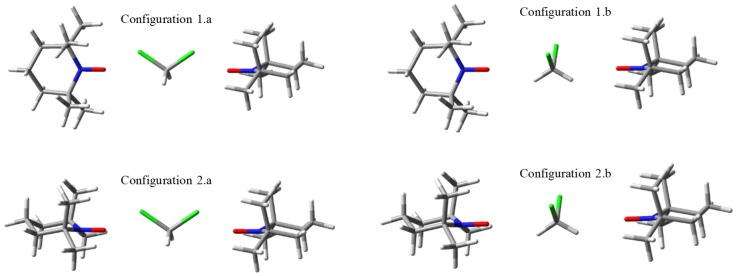
Scheme of molecular orientations in complexes including a dichloromethane molecule between two TEMPO radicals. The green color represents the chlorine atom and the other colors are the same as in the Figure 1.

For the sake of a further discussion concerning the role of solvent-mediated exchange coupling, taking dichloromethane as case study, Figure 2 presents the convened orientations of the TEMPO…CH_2_Cl_2_…TEMPO complexes. As we do not debate the solvation energy, the configurations are conventionally chosen to reflect distinct idealized interaction channels, eluding the aspects that emerge from the thermodynamic and kinetic factors, and without performing geometry optimization in these complexes. The carbon atom of the solvent is placed on the NO…ON axis, imposing two orientations of the solvent. The case labelled by ‘a’ enforces the chlorine atoms nearby the oxygen atoms of the TEMPO molecules, while the ‘b’ version brings the hydrogen atoms of the solvent in this situation. Consequently, adding these suffixes to the previous configuration names, we have coplanar CCl_2_ and NO…Cl moieties in the 1.a and 2.a geometries, while in 1.b and 2.b the planarity is imposed between the CH_2_ and NO…H fragments. 

The magnetic coupling between the TEMPO units is expected to strongly vary with generalized geometrical degrees of freedom of the interplayed solvent molecule, e.g., with its rotation, once kept a given centre of gravity, but we cannot account here for all of the possible structural variations, aiming to a certain simplicity of the conclusions, based on the above selected situations.

### 2.2. Orbital-Overlap Rationalization of the Exchange Coupling in Nitroxide-Based Radical Pairs

The fruitful orbital paradigms in the molecular magnetism [35,36] are based on the dichotomization of exchange parameter into a ferromagnetic part, always being positive due to pure exchange integrals, and a negative antiferromagnetic component, driven by orbital overlapping and other one-electron effects:

(1) $$J=J_{F}+J_{AF}.$$

In the case of the simplest exchange situation, namely two electrons placed in two orbitals on different sites, *a* and *b*, the pure exchange is ascribed as the following integral:

(2) $$J_{F}=\left(ab|ba\right)=\int \int a\left(1\right)b\left(2\right)\frac{1}{r_{12}}b\left(1\right)a\left(2\right)dV_{1}dV_{2},$$

where the labels 1 and 2 are referring to the generic different electrons, while the antiferromagnetic part can be effectively formulated as proportional to the square of overlap integral [52,53,54,55],

(3) $$J_{AF}=-k_{ab}S_{ab}^{2},$$

letting here the $k_{ab}$ factor without an explicit definition, the overlap integral having the following definition:

(4) $$S_{ab}=\int a\left(1\right)b\left(1\right)dV_{1}.$$

Although many other elements can contribute to the $J_{AF}$ part of the *a*-*b* inter-centre coupling, for instance, terms, like $h_{ab}^{2}$ or $h_{ab}\cdot S_{ab}$, (where $h_{ab}$ is a one-electron integral over kinetic energy and electron-nuclei electrostatics), their dependence on angular parameters of the *a* and *b* mutual orientation of the orbitals is the same like for the $S_{ab}^{2}$. Therefore, all the components, acting by a second-order perturbation theory can be ascribed in the above effective form. For the situation of exchange between the centres with more electrons, each *J_F_* and *J_AF_* is conceived as a sum over the different *a*-*b* orbital interaction channels. A plethora of computational data regarding exchange coupling in transition metal complexes were rationalized in the key of such heuristic models [56,57,58,59,60,61]. 

The Broken-Symmetry (BS) [62,63,64,65] is the method that is available and often used to estimate the exchange coupling parameters in the frame of DFT methods. This kind of calculation implies the “breaking” of the spatial symmetries by mixing the HOMO (Highest Occupied Molecular Orbital) with LUMO (Lowest Unoccupied Molecular Orbital). It is important to specify that the BS configurations are not real states. The BS approach is very often applied in the binuclear species, when the formula used to calculate the *J* coupling parameter is the one that was put down by Yamaguchi and Onishi [66,67]:

(5) $$J=-\frac{E_{HS}-E_{BS}}{\langle S^{2}\rangle {}_{HS}-\langle S^{2}\rangle {}_{BS}},$$

where HS denotes the High Spin configuration (the state with all electrons parallel and maximal spin) and BS stands for the Broken-Symmetry spin configuration (with different spin polarizations among centres and formal multiplicity corresponding to $\left|S_{a}-S_{b}\right|$). The formula is easy to use, because the *E* and $\langle S^{2}\rangle$ values are printed-out by the regular output of the ab initio packages. 

The geometry labelled as Configuration 1 imposes mutual perpendicular mean planes of the two {C_5_N} rings, therefore leading to orthogonal p-type lobes at the intermolecular interaction and consequently to null π-type overlap between the oxygen atoms. Conversely, in the Configuration 2 is expected that the unpaired electrons that are lodged in the π* antibonding molecular orbital (MO) of the NO moiety are realizing mutually a π-π type of long-range overlapping between the atomic orbitals (AOs) located on the oxygen atom. Subsequently, according to the basic knowledge from molecular magnetism [35,36], one may expect a ferromagnetic system in the first case, determined by the orbital orthogonality, while, an antiferromagnetic encounter in the Configuration 2, driven by the non-null overlap. 

Indeed, numeric experiments that were realized by BS-DFT treatments are yielding the expected sign of the exchange coupling parameter *J*. The calculations are performed with CAM-B3LYP functional [68] and the 6-311+G* basis. The positive exchange coupling values of the Configuration 1 are varying abruptly with the intermolecular separation, being satisfactorily fitted with a single exponential:

(6) $$J_{\left[1\right]}\left(r\right)=j_{\left[1\right]}^{0}\cdot \exp\left(-a_{\left[1\right]}\cdot r\right).$$

The Configuration 1 is assignable to the ferromagnetic component, *J*_[1]_(*r*) ≡ *J_F_*(*r*), with the corresponding parameters being: $j_{F}^{0}\equiv j_{\left[1\right]}^{0}$ = 7663.86 ± 239.5 (3.125%) cm^−1^ and *a_F_*≡ *a*_[1]_ = 2.812 ± 0.01 (0.3715%) Å^−1^.

Similarly, Configuration 2 can also be fitted with a single exponential, with $j_{\left[2\right]}^{0}$ = −193935 ± 13520 (6.97%) cm^−1^ and *a*_[2]_ = 3.112 ± 0.023 (0.75%) Å^−1^, the whole *J*_[2]_(*r*) curve being therefore in the negative domain. At the same time, this case can be regarded as having the exchange resulting as the sum of the ferromagnetic part and the maximal absolute value of the antiferromagnetic component ($J_{AF}^{0})$, which dominates in the resulting negative sign, namely $J_{\left[2\right]}=J_{F}+J_{AF}^{0}$. This situation corresponds to the maximal overlap $\left(S_{ab}^{0}\right)$ between the orbitals located on the oxygen atoms, in a π-π type long-range interaction. One may ascribe:

(7) $$J_{AF}^{0}=-k_{ab}\max(S_{ab}^{2})=-k_{ab}{\left(S_{ab}^{0}\right)}^{2}.$$

**Figure 3:**
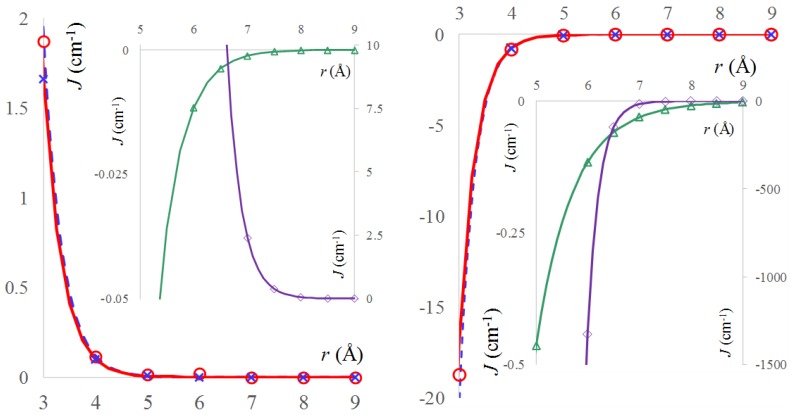
The computed and fitted curves for the variation of exchange coupling, *J*(*r*), as function of oxygen-oxygen distance (*r*) between two TEMPO radicals, taken in the Configuration 1 (left side) and Configuration 2 (right side). The circle symbols (ο) correspond to computed BS-DFT data, the continuous red lines represent the fitted exponential dependences. The (×) symbol stands for similar data, computed with Broken-Symmetry-Density Functional Theory (BS-DFT) data and Self Consistent Reaction Field (SCRF) solvent model, while the dashed blue lines are the corresponding fit. The insets show the results of BS-DFT calculations with true solvent molecules placed at conventional orientations between radicals (See Figure 2). The diamond symbols and violet lines correspond respectively to computed and fitted data in the case of a solvent arranged with the CCl_2_ moiety in the same plane with the NO…Cl ones; the *J* values are represented on the right-side abscissa of each inset. The triangle symbols and green lines represent the coplanar CH_2_ and NO...H moieties; the representation refers to the left-side abscissas of the insets.

Afterwards, an alternative way to treat the Configuration 2 is to subtract the values that were evaluated for ferromagnetic parameter, fitting by single exponential the antiferromagnetic part. Because of the net preponderance of the last term, the parameters for the $J_{AF}^{0}$ part are close to those of the whole curve of Configuration 2, having: $j_{AF}^{0}$ = −193496 ± 11280 (5.83%) cm^−1^ and *a*_[2]_ = 3.080 ± 0.023 (0.63%) Å^−1^. The results of BS calculations are shown in Figure 3 as marked points, the corresponding fitting being drawn with continuous lines. 

Figure 3 also includes the calculations and fitted results corresponding to solvent effects. The main graphs show, with dashed lines, the solvent added “from keyboard”, through the Self Consistent Reaction Field (SCRF) approximation, as implemented in the Gaussian09 program [69], testing here the built-in parameters for dichloromethane. As is visible, the computed points and fitted lines for the radicals in emulated solvent environment are not much departed from those resulted in vacuum. The insets show the exchange coupling computed when the solvent is added as a real CH_2_Cl_2_ molecule, being placed in between the NO groups. This variation will be discussed later on.

For a general geometry, keeping the colinear NO groups while rotating one TEMPO molecule around this axis, to obtain the α angle between the lobes of the p-type orbitals (as sketched in the middle of the Figure 1), the overlap integral varies as $S_{ab}=S_{ab}^{0}\cdot \cos\left(\alpha \right)$. Afterwards, at a given *r* separation between the oxygen atoms, the exchange integral is expected to vary with the α angle according to the following regularity:

(8) $$J\left(r,\alpha \right)=J_{F}\left(r\right)+J_{AF}^{0}\left(r\right)\cos{\left(\alpha \right)}^{2}$$

**Figure 4:**
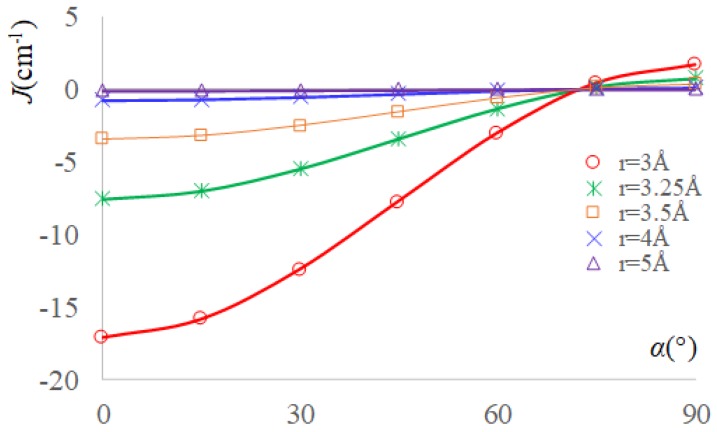
The exchange integrals as function of the α angle (see the scheme from Figure 1) between the p-type lobes in case of a TEMPO dimer with coaxial NO groups. The marked points correspond to BS-DFT computed data, the continuous lines being emulated with the Equation (8), based on the previously fitted $J_{F}\left(r\right)$ and $J_{AF}^{0}\left(r\right)$ parameters (red line for *r* = 3 Å, green line for *r* = 3.25 Å, orange line for *r* = 3.5 Å, blue line for *r* = 4 Å, purple line for *r* = 5 Å). Note that the representation does not imply an underlying fitting procedure.

The curves that are drawn in the Figure 4 are showing a perfect match between the computed values, which are represented as marked symbols, and the lines emulated with the above formula, based on the previously obtained $J_{F}\left(r\right)$ and $J_{AF}^{0}\left(r\right)$ dependences, without implying any new fit. If rescale the margins of each J(r,α) vs. α curve to the same graphical points, then the curves for different *r* values are looking the same. The absolute maximal and minimal values of *J* are rapidly dropping with *r*.

Very interesting conclusions are reached repeating the numerical experiment of TEMPO group rotation, while CH_2_Cl_2_ solvent molecules are placed in between, in the conventional ‘a’ and ‘b’ type positions that are suggested in Figure 2. The variation of α from 0° to 90° brings the Configuration 2.a into Configuration 1.a and Configuration 2.b into Configuration 1.b. The BS-DFT calculations with varying α should be carried out at larger separation between NO groups, at *r* ≡ *r*_OO_ ≥ 5 Å because of the bulk solvent molecule. Although at these distances the pristine couple of radicals shows practically null spin coupling, the bridged systems are presenting large magnification for situation ‘a’ and sizeable values for the ‘b’ cases. 

**Figure 5:**
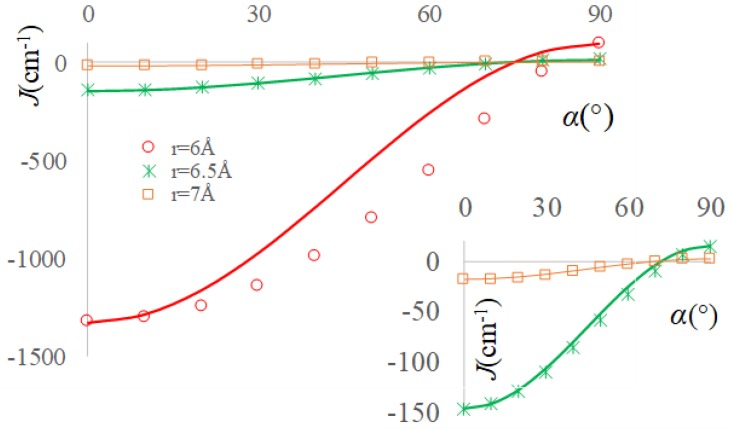
The exchange integrals as function of the α angle for ‘a’-type the TEMPO…CH_2_Cl_2_…TEMPO complexes. The marked points correspond to BS-DFT computed data, the continuous lines being emulated with the Equation (8), according to the text discussion. The inset shows magnified representation of the curves at *r* = 6.5 Å and *r* = 7 Å separation of oxygen atoms from radicals. The red line is the fit for *r* = 6 Å, the green line is for *r* = 6.5 Å and the orange line is for *r* = 7 Å.

**Figure 6:**
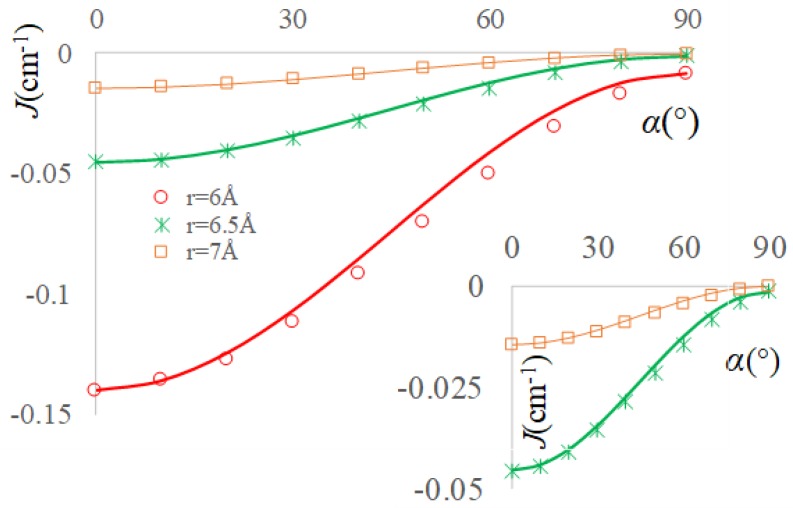
The exchange integrals as function of the α angle for ‘b’-type the TEMPO…CH_2_Cl_2_…TEMPO complexes. The content is similar to the representation from Figure 5.

Somewhat intriguingly, it is observed from Figure 5 and Figure 6, corresponding to respective ‘a’ and ‘b’ systems, are roughly retaining the pattern recorded in Figure 4. The deviations are larger at the smaller *r* = 6 Å, while they tend to be almost ideal for the *r* = 6.5 Å and *r* = 7 Å (i.e., with the curve given by Equation (8) passing through the computed points). The biggest irregularity is seen in the ‘a’ case, at *r* = 6 Å. In the ′av situation, the α = 90° structures are yielding positive *J* values, as in the ideal orthogonality situation, although the intervening solvent might change the validity of overlap-based rationalization. 

Enforcing the consideration of α = 90° and α = 0° margins as corresponding to the $J_{F}\left(r\right)\;\mathrm{and}$ $J_{AF}^{0}\left(r\right)$ in the case of solvent mediated exchange, one might generate the curves by Equation (8). As clearly visible in Figure 5, at small separation of radicals, the “lensing” effect of the solvent results in large enhancement of all the *J* values and a certain deviation from the idealization by Equation (8). At larger radii, as seen in the inset of Figure 5, the validity of approximation by the Equation (8) is surprisingly restored, undergoing also a large boosting of absolute *J* values, in comparison with the corresponding case with no solvent in between. 

In the ‘b’ series, the *J* coupling parameters are raised, in comparison to the through-vacuum exchange, but much lesser, by four orders of magnitude, when compared with the ‘a’-type systems. The interpolation by Equation (8) is well working at the *r* = 6 Å points and almost perfectly for the *r* = 6.5 Å and *r* = 7 Å ones. In the ‘a’ cases, the α = 90° coupling is not positive, having small negative or null value. In the circumstance, it is only enforcedly taken as the *J*_F_ component, but ignoring this shift, the angular correlation works, as noticed. 

The fact that regularities emerging from qualitative orbital overlap rationalization (from where the angular dependence comes) are kept in the case of through-solvent interactions is quite unexpected, which encourages the idea that magneto-structural correlations can be extended to complicate and dynamic media. This is just an incentive point, since the way to the full realization of such desiderata is long, demanding a lot of construction, by further numeric experiments. The radial dependence of the solvent-mediated exchange coupling is discussed in the next section. 

### 2.3. The Real Solvent Effects in Long-Range Exchange Coupling

The insets that are given in the Figure 3 show that the solvent strongly alters the exchange coupling between the NO radicals. The ‘a’-type configurations show a strong magnification of the coupling intensity, keeping the ferromagnetic result in the case of 1.a and the antiferromagnetic nature for the 2.a geometry. Due to of steric hindrance, the admissible oxygen—oxygen distances must be larger than about 5 Å. To illustrate the enhancement, let us pick the following comparisons: the exchange at *r* = 7 is *J* = 2.692 cm^−1^ in the 1.a solvent case, definitely higher than the *J* ~ 0 cm^−1^ for the simple Configuration 1. At *r* = 7, Configuration 2 yields *J* = −18.712 cm^−1^ with solvent, much more antiferromagnetic than the *J* = −2 × 10^−4^ cm^−1^ value for the through-space coupling. The contribution of the solvent can be fitted also with single exponential curves, after extracting the curve adjusted for the pristine TEMPO dimers. Placing the solvent in a configuration labelled by the ‘a’ suffix, one finds a smaller enhancement of the ferromagnetic interaction in Configuration 2, while Configuration 1 is turned from ferromagnetic to antiferromagnetic. 

**Figure 7:**
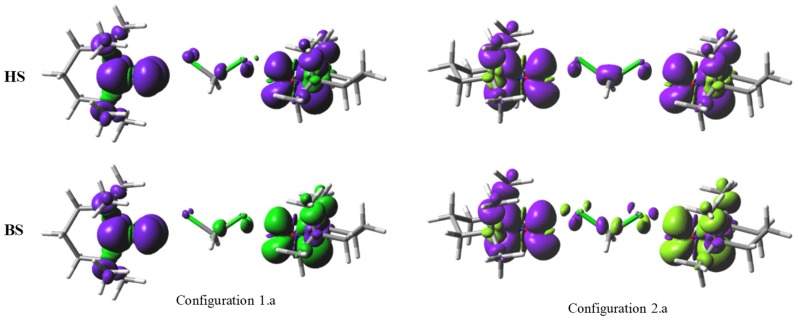
The spin density maps, as 0.001 e/Å^3^ isosurfaces, for the 1.a and 2.a configurations, at the *r* = 7 Å separation between the oxygen atoms of the colinear NO groups, with the oxygen atoms in the vicinity of the chlorine atoms of the dichloromethane molecule. The upper part shows the predominant α density of the triplet configuration, labelled HS (High Spin). The lower half shows the Broken Symmetry (BS) configuration. The violet and green colours correspond to the respective α and β spin densities.

Let us analyse the spin density distribution and the spin density difference maps in the computed complexes, after subtracting the clouds of the standalone solvent and those of the TEMPO dimers to understand the mechanisms of mediation exerted by the solvent (as converged in the through-space interaction cases). The spin density maps from the Figure 7 show the active role of the solvent, involved in the polarization of α and β clouds, in the case of the ‘a’-type placement of the CH_2_Cl_2_ molecule. The displayed configuration corresponds to a separation by 7 Å of the oxygen atoms from the two TEMPO molecules, resulting in about 2.26 Å as oxygen-chloride distance, when the carbon atom of the solvent is kept on the O…O line. This geometry is conventionally selected, as a case of undergoing a sizeable exchange interaction. One observes that the CH_2_Cl_2_ molecule carries a partial spin density, in both HS and BS cases, in the 1.a and 2.a configurations. In the HS cases, the overall colouring is rather uniform, corresponding to the net domination of the α spin density. 

The partial density over the solvent suggests the propensity of the CCl_2_ moiety to transmit the exchange coupling, by delocalization through the virtual orbitals ported by the chlorine atoms, in spite of the fact that the Cl…O contacts are not favourable from energy point of view. The conceivable mechanism is a donor-acceptor relation of the respective oxygen-chlorine contacts. Qualitatively translating the discussion in terms resembling the restricted-open shell (although, technically, the unrestricted frame was used), the donors are the single occupied molecular orbitals (SOMOs) of each TEMPO group, while the acceptors are virtuals belonging to the chlorine atom. 

In the particular geometry of the Configuration 1.a, the Cl-C-Cl moiety, as bridge of mediated coupling, the conserves orthogonality of magnetic orbitals, which now are showing tails on the chlorine atoms. In the left-upper corner of Figure 7, one observes that the right-side monomer has the residual spin-density of the chlorine in the C-Cl...O plane, while in the left-side part it occurs perpendicularly to the corresponding O...Cl-C plane. Therefore, the two halves of the Cl-C-Cl sequence are in the orthogonality relationship, exhibiting the ferromagnetic coupling, enhanced with respect of the through-space interaction of the nitro groups, because of the new orbital channels that were opened by the donor-acceptor processes. In the case of Configuration 2.a, the interaction of both monomers induced the spin density delocalization (see the HS map, right-upper corner of Figure 7) and spin polarization (see the BS map, right-bottom corner of Figure 7) in a plane common for the O…Cl-C-Cl…O fragment, the diffuse orbitals that are available at chlorine determining the strong antiferromagnetism. 

**Figure 8:**
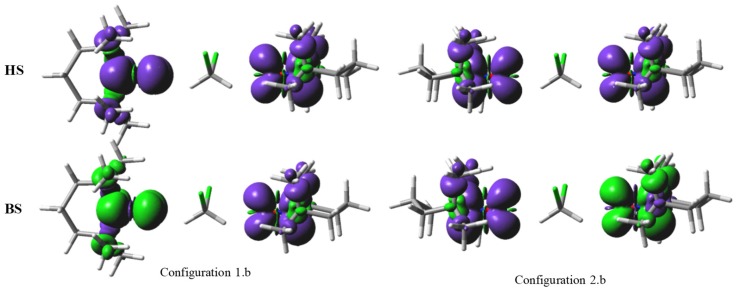
The spin density maps, as 0.001 e/Å^3^ contours, for the 1.b and 2.b configurations, at the *r* = 6 Å separation between the oxygen atoms of the colinear NO groups, with the oxygen atoms in the vicinity of the hydrogen atoms of the dichloromethane molecule. The notations and colouring are similar to those from Figure 7.

Figure 8, devoted to the ‘b’-type geometries, shows that, in this placement, the solvent molecule is little responsive to the spin density of the neighbouring radicals (it shows residual tails only at lower thresholds). However, the interposed molecule affects the exchange, causing a certain amplification of the antiferromagnetism in the case of the 2.b configuration. For instance, at *r* = 6 Å, the *J* parameter for the TEMPO dimer is almost negligible, −0.003 cm^−1^, while sizeable, *J* = −0.116 cm^−1^, for the TEMPO-CH_2_Cl_2_-TEMPO complex in the 2.b geometry. A puzzling turn is recorded for Configuration 1.b, since the ferromagnetic nature of the TEMPO couple in Configuration 1 is now transmuted in the antiferromagnetic coupling. The moderate enhancement of antiferromagnetism in Configuration 2 and its switch to antiferro coupling in the Configuration 1 can be explained by the fact that, although the HCH fragment of the solvent comes in closer contact with the radicals, the effective orbital mediation of the interaction is ensured, in long-range regime, also by the ClCCl fragment. The CH bonds are tightly holding their density, less polarizable, and poor in atomic orbital components available for donor-acceptor effects. In turn, even more distant, the rich orbital “atmosphere” of the chlorine atoms, can intercept and transmit the spin signals between the two radicals. In the ‘b’-type geometry, the C-Cl bonds are available for a simultaneous interaction with both TEMPO radicals, irrespective their mutual placement. Subsequently, their intervention in the interaction tableau annihilates the premises of ferromagnetism inferred in the case of orthogonality relationship between the TEMPO units, themselves. 

In the end, we will add a brief test for the situation of radicals interacting over two solvent molecules. We will conventionally consider a situation resembling the Configuration 2.a. Namely, we will take one TEMPO and one CH_2_Cl_2_ molecule with a 3 Å separation between the oxygen atoms of the radical and the carbon of the solvent. Then, this couple is replicated over an inversion centre placed at 2 Å from the carbon, in the opposite side of the TEMPO unit. This results in TEMPO…CH_2_Cl_2_… CH_2_Cl_2_…TEMPO sequence having the solvent molecules distanced by 4 Å between their carbon centres. The oxygen-oxygen overall distance is 10 Å. With respect of radical-solvent spacing, this situation is akin to the Configuration 2.a taken at 6 Å oxygen-oxygen distance, which shows a strong antiferromagnetic coupling of about −1500 cm^−1^. The new two-solvent configuration yields −16.25 cm^−1^. This is remarkable interaction at distance, when considering that, in the Configuration 2.a taken at 10 Å inter-radical separation, the *J* parameter is practically vanishing. As in the previous instances, the chosen configuration is a purely hypothetical one, the inversion centre between the solvents was taken in the idea of a prefered antiparalell alignment of their electric dipoles, but otherwise we neglect the details of total interaction energy and optimal geometry. We retain the idea that, in certain situations, the spin coupling can be transmitted via solvent at very long range. The aspects that are related with the energetics of TEMPO in media of halogenated solvents are debated elsewhere [70,71].

The bold conclusion is that the SCRF cannot act as a salient surrogate in BS-DFT simulations of the radicals in a solvent medium. The SCRF is designed to describe global inter-molecular energy effects, being unable to deal with the fine details that are involved in the exchange coupling mechanisms. The BS-DFT calculations suggest that, as a function of the solvent nature and its placement in between the interacting radicals, the exchange coupling can be dramatically changed, in sign and magnitude, in comparison to the pristine couples that were taken in vacuum. Probably, with a dedicated effort, models resembling the superexchange [72] in ligand-bridged metal ions in coordination polynuclears can be conceived for the role of solvent. Of course, these would very specifically depend on the given solvent. The subject is too vast to be resolved here, which limits our role to point the finger on this uncharted area in the landscape of molecular magnetism ported to a solvent medium. Although difficult and somewhat cumbersome, it is challenging to aim at understanding the specific orbital factors of various solvents, as function of their orientation towards the neighbouring spin carriers. 

### 2.4. Dipolar Coupling, Zero Field Splitting and Solvent Effects

In the following, we will take computational experiments that question the issue of magnetic dipole-dipole coupling between pairs of TEMPO radicals, with and without solvent molecules in between. The rationalization of this field is a valuable attempt, when considering that the corresponding parameters are useful in the practice of EPR spectroscopy as spin-probe tool [1,28,29,30,31,32,33,34].

Starting with all the pedantic details, let us ascribe the general form of the so-called Zero-Field Splitting (ZFS) Hamiltonian [35]:

(9) $${\hat{H}}_{\mathrm{ZFS}}=D\left({\hat{S}}_{z}^{2}-\frac{1}{3}S\left(S+1\right)\right)+E\left({\hat{S}}_{x}^{2}-{\hat{S}}_{y}^{2}\right).$$

It can manifest on spin states with *S* ≥ 1, leading, as its etymology says, to gaps inside the multiplets that are established by the corresponding *S_z_* sets (otherwise degenerate in the absence of an external magnetic field). It has different sources, like spin-orbit coupling in transition metal complexes [73], or the dipolar interactions in the couples of organic radicals [74], as will be the case in our following discussion. In the International System of Units, the interaction energy between two magnetic moments, as defined by the µ*_A_* and µ*_B_* vectors, is:

(10) $$E_{AB}=\frac{1}{4\pi \varepsilon c^{2}}\left(\frac{\mu_{A}\cdot \mu_{B}}{r_{AB}^{3}}-3\frac{\left(\mu_{A}\cdot r_{AB}\right)\left(\mu_{B}\cdot r_{AB}\right)}{r_{AB}^{5}}\right),$$

where **r***_AB_* and *r_AB_* are the vector and length that correspond to the line between *A* and *B* centres. Obviously, *c* and ε are the speed of light and permeability of vacuum. Introducing the quantum operators, i.e., the $\mu =g\beta \hat{S}$ vector and the $\mu =g\beta \sqrt{S\left(S+1\right)}$ module, where β is the Bohr magneton and *g* is the isotropic Landé factor, one finds the following form of the dipolar Hamiltonian [38]:

(11) $${\hat{H}}_{dip}=D_{AB}\left({\hat{S}}_{z_{A}}\cdot {\hat{S}}_{z_{B}}-\frac{1}{3}{\hat{S}}_{A}\cdot {\hat{S}}_{B}\right).$$

The leading parameter corresponds to the following expectation value with respect of the *Ψ* wavefunction:

(12) $$D_{AB}=-\frac{9}{4}\cdot \frac{g^{2}\beta^{2}}{4\pi \varepsilon c^{2}}\langle \Psi |\frac{1}{r_{AB}^{3}}|\Psi \rangle ,$$

which turns to the following integral,

(13) $$D_{AB}\propto -\int \int \sigma_{A}\left(r_{A}\right)\frac{1}{r_{AB}^{3}}\sigma_{B}\left(r_{B}\right)dr_{A}dr_{B},$$

in terms of σ spin densities around the distinct radical molecules *A* and *B*.

We are concerned with the ZFS on the *S* = 1 state that resulted from the coupling of two nitroxide-type radicals. If the spin momenta can be idealized in point-like localizations, the relation between the *D* and *D_AB_* parameters from the above (9) and (10) formulas is

(14) $$D=-\frac{1}{2}\left|D_{AB}\right|.$$

If aim for *D_AB_* in reciprocal centimetre (cm^−1^) as units, while using Angstrom (Å) as length measure, we have

(15) $$|D_{AB}|=1.299\frac{g^{2}}{r_{AB}^{3}},$$

while the *E* parameter is null. The Landé factor can be fairly taken almost *g*~2 (i.e., close to the free electron value *g*_0_ = 2.0023). However, when the spin has a certain space distribution, the above relation might not be straightforward, and an effective *E*-type ZFS parameter may appear.

**Figure 9:**
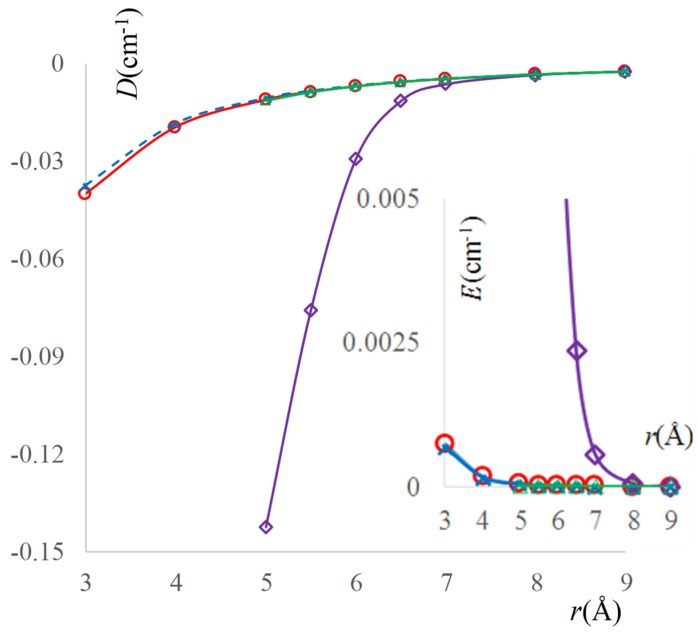
The Zero Field Splitting (ZFS) parameters computed for Configuration 2, as function of separation between the oxygen atoms of the colinear NO groups. The main panel shows the variation of the *D* parameter for: two TEMPO molecules in vacuum (red line and open circles); with SCRF solvent option (dashed light blue lines and × symbols); two TEMPO molecules with a CH_2_Cl_2_ solvent in between, having the CCl_2_ and NO…Cl sequences coplanar (Configuration 2.a-violet line and diamond symbols); two TEMPO molecules with solvent oriented with coplanar CH_2_ and NO…H parts (Configuration 2.b-green line and triangle symbols). The inset corresponds to the *E* parameter, drawn with the same conventions, for the respective symbols. The Configuration 1 shows a very similar variation of *D*, while the *E* is rigorously null, by symmetry reasons.

We are going to check the pattern of the ZFS parameters by computation, taking the same molecular models from the previous section, namely a pair of TEMPO radicals, interacting through-space or via a dichloromethane molecule. The results, produced with Orca program [75], B3LYP functional, and EPR-II basis are shown in Figure 9, while considering Configuration 2 of two TEMPO molecules. One notes that the calculation with implicit SCRF-type solvent gives almost the same results as the calculation for the molecules in vacuum. The curve for the system with a solvent intruding with the CH_2_ plane as bridge between the NO group (Configuration 2.b) is closely similar to the above curves, in the part that is allowed by steric hindrance (separations between oxygen atoms larger than 5 Å). In turn, when the CCl_2_ plane of the solvent is offered as interaction path (Configuration 2.a), the change is drastic at smaller distances between radicals, while at larger radii the asymptotic trend to zero becomes like in the other cases. This system also shows a pattern of *E* parameter differing from those of the other three systems, which are mutually similar. This can be explained on the same grounds discussed at the check of exchange coupling dependence in the same model systems. The diffuse virtual orbitals that are available at the chlorine are making this part of molecule a “non-innocent” actor in the intermediation of spin interactions. A good part of spin density is delocalized over the interposed solvent unit, sensibly altering the long-range nature of the interaction. *Sed contra*, the CH_2_ half has low electronic density, tightly confined around the proton centres, with little susceptibility for transmitting spin effects, so that in this case the solvent is practically transparent to the dipolar coupling. 

The calculation of the *D* parameter on Configuration 1 of TEMPO units yield closely similar results with Configuration 2, in the following instances: systems in vacuum, under SCRF treatment, and in the case of NO...HCH...ON bridged system. The system with NO…ClCCl…ON arrangement yields a similar pattern, with slight numerical differences. For instance, at distant points, with *r*_OO_ = 8 Å and 9 Å, the respective values for Configuration 1 are *D* = 3.34 × 10^−3^ cm^−1^ and *D* = 2.40 × 10^−3^ cm^−1^, while Configuration 2 has *D* = 3.47 × 10^−3^ cm^−1^ and *D* = 2.42 × 10^−3^ cm^−1^. At shorter contacts, *r*_OO_ = 5 Å and 5.5 Å, the departure is larger, with *D* = −0.1390 cm^−1^ and, respectively, *D* = −0.0418 cm^−1^ for Configuration 1, while *D* = −0.1426 cm^−1^ and *D* = −0.0759 cm^−1^ for Configuration 2. 

The absolute values of *E* are small in Configuration 2, with steep decay at larger separations, are null, by symmetry reasons, in Configuration 1 without solvent or with SCRF solvation. The *E* values are very small, in the 10^−5^ −10^−7^ cm^−1^ range, in the Configuration 1 with NO...HCH...ON constitution. Although, from geometrical point of view, the inserted molecule destroys the axial symmetry expected in ZFS effect, it is safeguarded effectively by the low responsivity of this molecular arrangement. The CH_2_Cl_2_ breaks the symmetry of the formal spin arrangement of Configuration 1, leading at *r*_OO_ = 5 Å to *E* = 8.48 × 10^−2^ cm^−1^, close to the *E* = 8.89 × 10^−2^ cm^−1^ value of the Configuration 2 in the same interaction conjuncture. Besides, the *E* value less suddenly drops with distance in the case of Configuration 1, being, for instance, *E* = 5.75 × 10^−2^ cm^−1^ at *r*_OO_ = 5.5 Å, while the Configuration 2 gives *E* = 2.35 × 10^−2^ cm^−1^ for the same separation of the oxygen-oxygen ends. 

The intervention of the *E* parameter is not considered in the EPR spectroscopy of the nitroxide-based radical couples, the small values retrieved from calculations justifying this practice. However, we may inquire about its physical origin. It is related with the deviation of the spin density from an axial pattern. Actually, the data computed for the above discussed systems in vacuum can be fitted satisfactorily with a rule of third inverse power between the middles of the NO bonds, or, in other words, taking the Equation (14) with *r*_AB_ = *r*_OO_ + *d*_NO_, i.e., the NO bond length added to the O…O linear separation. Especially, at large separations, the radical character distributed on the NO group can be regarded under the point-wise approximation. At lower radii, the details of the spin map start to count, this being the mechanism for the apparition of the *E* parameter. 

The computed *D* and *E* parameters can be perfectly fitted with a model considering the spin of a TEMPO unit distributed in the four points, assignable to the barycentres of the p-type lobes making the antibonding π* orbital hosting the spin density on the NO group. Thus, with the assistance of chemical intuition, one might propose the replacement of the integral from Equation (14) by a finite summation over the representative points schematizing the charge distribution on subsystems *A* and *B*:

(16) $$D_{AB}=-1.299g^{2}\sum_{i=1}^{N_{A}}\sum_{j=1}^{N_{B}}\frac{\sigma_{A}\left(r_{A_{i}}\right)\sigma_{B}\left(r_{B_{j}}\right)}{r_{A_{i}B_{j}}^{3}}.$$

**Figure 10:**
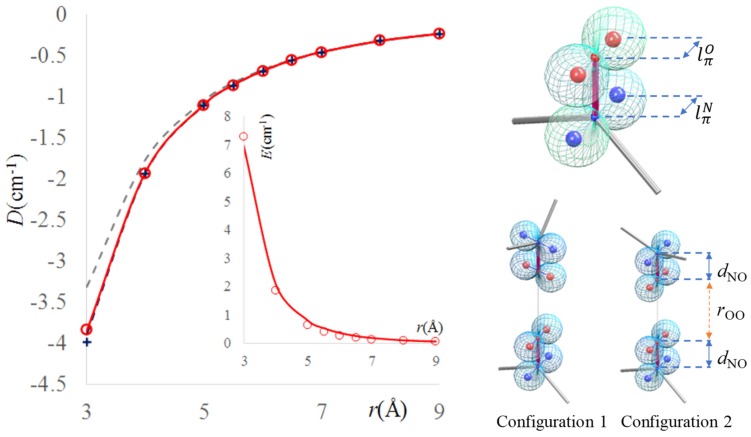
The modeling of ZFS parameters. Left panel: the dashed grey line estimated *D* parameter, assuming point-like spins in the middle of the NO groups (approximation common for Configurations 1 and 2); the plus (+) and open circles (o) symbols correspond to computed *D* values for the respective Configuration 1 and 2. The lines passing through these points correspond to the fit with the model sketched in the right-side panel. The inset is dedicated to the *E* parameter, computed (circle symbols) and modelled (line), for Configuration 2, the respective values being null for the Configuration 1. Right-side panel: in upper part, the model with four points carrying spin density, in the barycentre of p-type lobes of oxygen and nitrogen (red and blue spheres); the bottom part shows the situations corresponding to Configurations 1 and 2; the TEMPO units are schematized by their C-NO-C moieties.

In the case of the TEMPO, the effective spin carriers can be taken on lines passing through N and O centres, simultaneously perpendicular to the N-O and C…C axes of a C-NO-C moiety of the molecule. We ascribe by $l_{\pi }^{N}$ and $l_{\pi }^{O}$ the distances from the N respective O sites to these spin barycentres, letting their values as object of the fit (see Figure 10). The best match was found for $l_{\pi }^{N}=$ 0.574 Å and $l_{\pi }^{O}=$ 0.461 Å. The magnetic momentum at each point is taken half of the Mulliken spin population on the respective nitrogen and oxygen atoms, while presuming that this does not vary from system to system (which is true, in a fair approximation). For a TEMPO molecule in unrestricted calculation, almost irrespective of detailed setting, one may consider the spin population of nitrogen about 0.45, while those of oxygen 0.51, the remainder from their sum, up to the unity, being distributed in traces over the neighbouring skeleton. Afterwards, neglecting the fine details, the above values are divided over the two π-type poles of each atom from NO. 

The above sketched four-point model very well accounts for the ab initio results of the ZFS parameters. However, a simplified two-point approximation (with spin thought located in the middle of NO bonds) might be satisfactory. The 1/*r*_AB_^3^ dependence is well kept in the case of radicals in vacuum and even in certain orientations of an inter-placed solvent molecule. Solvent placements that involve the closeness of atoms carrying diffuse orbitals (like chlorine in our case studies) may lead to a distortion from ideal variation of *D* parameter with distance, but with a relative lesser extent than was observed in the exchange coupling interaction.

### 2.5. The Basis Sets and the Hyperfine Coupling

We will close the computational experiments revisiting the parameters that are related with the EPR spectroscopy touching the problem of the hyperfine coupling [38,76,77]. For a general case of a set of nuclei, with components labelled by *i*, having each the electronic spin *S_i_* and the *I_i_* nuclear spins, the isotropic coupling is described by the *A_i_* parameters and the following phenomenological Hamiltonian:

(17) $${\hat{H}}_{\mathrm{Hyp}}=\underset{i}{\sum }A_{i}{\hat{S}}_{i}\cdot {\hat{I}}_{i}$$

Particularly important is the case of abundant ^14^N isotope, having a *I* = 1 nuclear moment. We skip the discussion regarding the *g* factor, since, in the case of stable nitroxide systems, it does not show spectacular variations, neither in experimental nor in computational respects, remaining almost isotropic and nearby the free electron value [10,37]. Despite being small in the energy scale, the computed hyperfine term is very sensitive to the basis set. Is a truism that all of the computed data depend on the method of choice and selected basis set, but the hyperfine coupling poses quite extreme challenges. Namely, it measures a coupling taking place at the nucleus (between the nuclear spin and the nearby spin density), being also sensitive to long-range effects, like the interactions with the solvent. 

Therefore, a basis set accounting for *A* parameters should be accurate both at nucleus and at the outskirts of the atom. Being concerned with the density in the range of the covalent or ionic radii, most of the bases may ignore the details at the nucleus. For this reason, in calculations with Gaussian type orbitals (GTOs), must use special bases, explicitly named EPR-II and EPR-III [78], when the account of the *A*-type parameters is envisaged. However, we can audaciously claim that practically none of the basis sets in use are taking a proper care of the long-range effects. 

With the occasion of a recent review on basis sets, we noticed that their long-range tails are tacitly and artificially attenuated [79]. This happens in the Gaussian-type orbitals because of the poor design of the $r^{k}\exp\left(-\zeta \cdot r^{2}\right)$ primitives, keeping the *k* powers strictly equal to the value of the secondary quantum number of the given atomic shell, *k* = *l*. A glance at the general structure of the exact atomic orbitals of the hydrogen atom might suggest that a shell described by the *n* and *l* quantum numbers implies a set of $r^{k}\exp\left(-\zeta \cdot r\right)$ Slater Type Orbital (STO) primitives, with *k* in the 0: *n*-1 range. For reasonable performance, it would be ideal for GTOs to use similar sets of radial cofactors in the $r^{k}\exp\left(-\zeta \cdot r^{2}\right)$ components. However, this is not happening. 

Although the bases can be considered just pragmatic objects, without knowing or inquiring about their relationship with a rigorous ideal, the limited radial cofactors are seriously impinging upon the flexibility of the technical utility. This issue remained hidden to most users of quantum chemistry, being a tribute paid to the saving of computing time in the very first codes, inherited as established routine, from generation to generation, until today. 

We will illustrate the issue of basis set and computed hyperfine coupling on the TEMPO molecule, while using the Amsterdam Density Functional (ADF) code [80,81], one of the very few programs working with Slater type orbitals (STOs). We opted for this because the ADF allows for a flexible customization of STO components, while it would be of tantamount difficulty to reshape the infrastructure of consecrated GTO codes, confining ourselves to launch a caveat about this sort of problem. Even in this circumstance, we tried to partially answer the challenge, tailoring ourselves a new basis, with satisfactory behavior over all the range: nucleus, valence, and beyond. 

**Figure 11:**
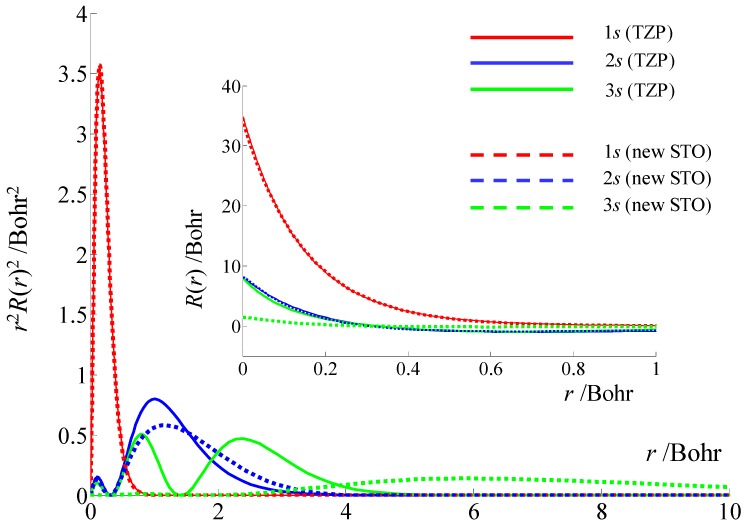
Radial profiles of the 1s, 2s, and 3s atomic orbitals of the nitrogen, computed with STO-type functions and BLYP (Becke-Lee-Yang-Parr) functional in the ADF code. The continuous lines correspond to the standard TZP basis, the dashed lines illustrating preliminary tests with a new basis showing long-range maxima in the *r*^2^*R*(*r*)^2^ functions. The inset shows the *r* vs. *R*(*r*) variation near nucleus.

The experimental *A* parameter for TEMPO is around 50 MHz, e.g., A = 50.7 in the reference [76] or 47.7 MHz in the [77] work. It depends on the solvent and other conditions, but in a somewhat moderate measure, because of the protective fence raised by the four surrounding methyl groups nearby the nitrogen atom. The estimation with the ADF code and the implemented triple zeta and polarization (TZP) basis set yields an underestimated value, of 31.7 MHz. The calculation taken with our new basis, allowing for virtual atomic orbitals with much ampler radii (see Figure 11), gives a better agreement, with 51.3 MHz. Must point that the basis is not fitted to produce better results for the isotropic factors. The primitives were fitted to the numeric atomic orbitals produced by the *atompaw* code [82], usually employed to produce pseudo-potentials for plane-wave methods. To be distinguished from TZP, which, for the s-type orbitals, has a set of two primitives with exp(−ζ⋅r) form and three others with rexp(−ζ⋅r), we considered a basis made of three simple exponentials, exp(−ζ⋅r), two like rexp(−ζ⋅r), and one with the $r^{2}\exp\left(-\zeta \cdot r\right)$ definition. The last term is helpful in acquiring the long-range profile for the atomic virtual orbitals. 

**Figure 12:**
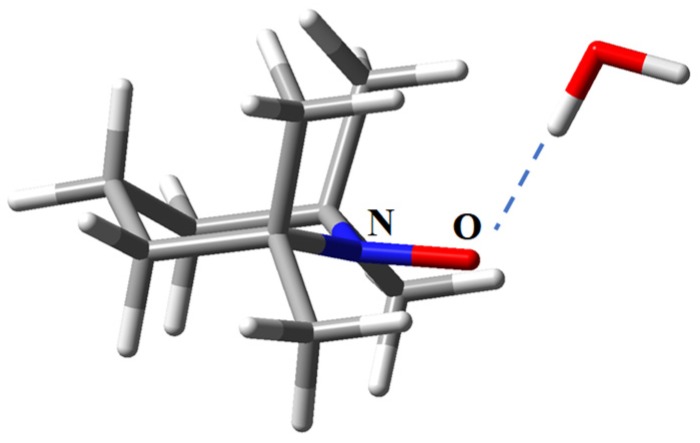
Hypothetical TEMPO…H_2_O complex used to test the response of computed hyperfine coupling to environment effects (by comparison with pristine TEMPO result).

To test responsivity to the environment, we took the model ensemble between TEMPO and a water molecule (see Figure 12). This was conventionally placed in the symmetry plane of the organic radical, realizing a NO…HOH hydrogen bonding, taking the geometry optimized under the *C_s_* point group. The effect is indirect, being propagated from oxygen’s vicinity to the nucleus of the nitrogen atom. The calculation with the standard TZP basis gives 34.9 MHz, while the new basis gives *A* = 60.3 MHz, i.e., a larger shift, in comparison with TEMPO standalone molecule. This can be correlated with the longer tails of the new basis, which are able to act as better “antennas” to the environment details. 

Figure 11 clearly illustrates that the tentatively new proposed basis shows for the 3s shell (in the *r* vs. *r*^2^*R*(*r*)^2^ representation) maxima beyond the atomic radius, at about 6 Bohr. The 3s in the standard TZP set of the ADF suite has an average radial extension close to those of the 2s, which is not quite reasonable. The 1s are very similar in the two bases, the 2s profiles having minor mutual differences. The 1s and 2s show similar curves when the two bases are compared in the *R*(*r*) function near nucleus. The new 3s, shown with dashed light green in the inset of Figure 11, has smaller value at *r* = 0 than the 3s from TZP (that here is accidentally coincident with the 2s curve). This is also reasonable, since larger orbits are supposed to have lower amplitudes at low radii. In other words, the density must be invested in the atomic periphery on the expense of lower representation in the inner part. We limited the discussion on the s-type orbitals, because having non-null density at nucleus are directly responsible for the Fermi contact phenomena that determine the isotropic hyperfine coupling. 

The calculations that were performed on the same systems with the Gaussian-type bases called EPR-II and EPR-III [78] are yielding the 29.9 MHz and 30.5 MHz respective values for the TEMPO in vacuum, while 32.9 MHz and 33.5 MHz for the TEMPO…HOH complex. These are similar to the TZP-based results with STOs from ADF. Paradoxically, Pople -type bases [83], rated with lower quality are giving seemingly better results. Thus, the 6-31G and 6-31+G are producing the 39.1 MHz and 39.5 MHz values for TEMPO, with 43.3 MHz and 43.4 MHz for its complex with water. The attempt to increase the basis quality in this series, going to 6-31+G*, turns into a lesser match, obtaining 34.1 MHz and 36.9 MHz for the compared couple of systems. This shows that the problem is a tricky one, our firm point being that the hidden trap is that the GTOs are lacking a proper leverage, provided by a desirable use of a general $r^{k}\exp\left(-\zeta \cdot r^{2}\right)$ series of primitives. According to the previous discussion, the s-type GTOs are only constructed with $\exp\left(-\zeta \cdot r^{2}\right)$ components, with no radial cofactors, at all. This makes very difficult the attaining of radial maxima beyond the atomic radii, a feature that is critical to the requirements of the hyperfine coupling calculations. 

We are not launching ourselves in the full critical debate and in the tedious duty of fabricating a new garniture of EPR-dedicated basis sets now. Aiming here for the larger picture of possibilities and challenges in topics related with the spin chemistry of nitroxide-type systems, we confine ourselves in pronouncing the caveat against the actual situation, while preparing the preliminaries for attempting the great challenge for a new generation of basis sets. 

### 2.6. Host-guest Assemblies with TEMPO-type Radicals 

**Figure 13:**
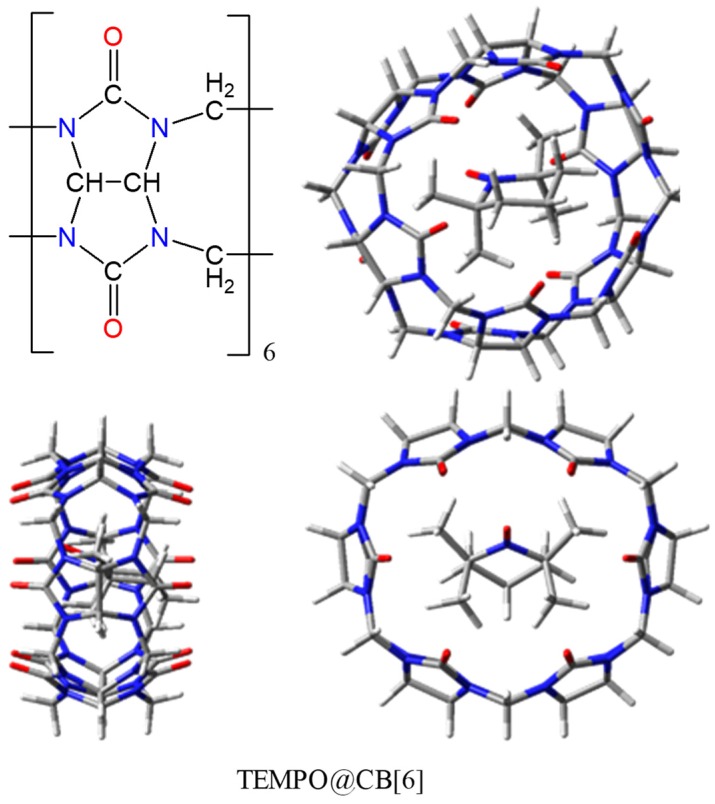
Chemical scheme of cucurbit[6]uril (CB[6]) host molecule and different views of optimized host-guest complex with TEMPO radical, TEMPO@CB[6]. The red color is for the O atom and the blue color is for the N.

**Figure 14:**
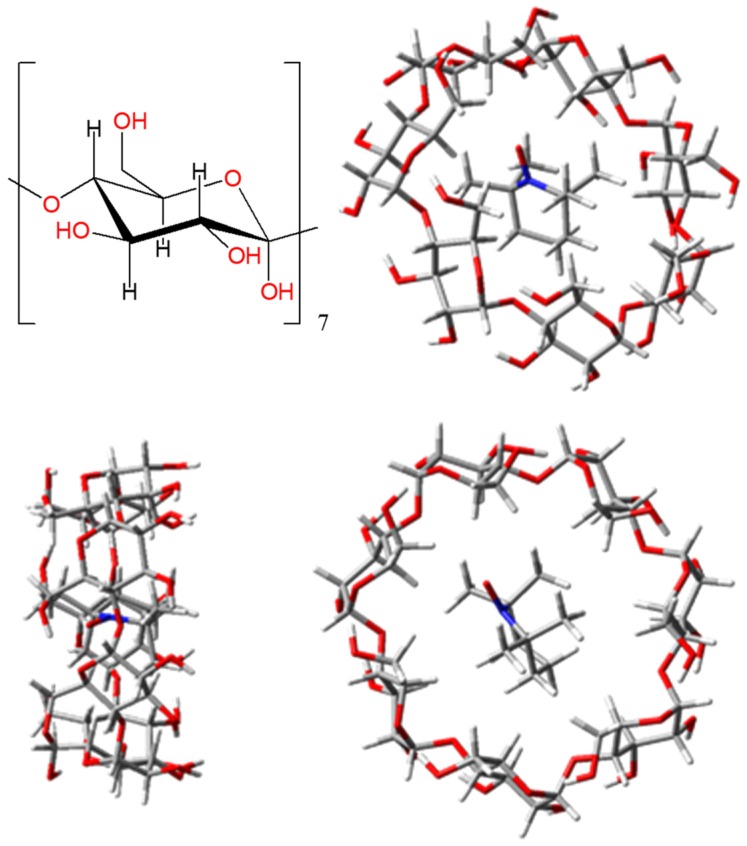
Chemical scheme of β-cyclodextrin (β-CD) host molecule and different views of optimized host-guest complex with TEMPO radical, TEMPO@β-CD. The color code is the same as in Figure 13.

An interesting topic in the physical chemistry of TEMPO radicals is the interaction with molecules that are able to act as encapsulating hosts. Although in technical respects the issues debated in the following are rather different from the focus of previous sections, the redline is based on the idea that host-guest interactions are important in the spin-probe use of EPR measurements [1,98,99]. Prototypic examples of host molecules are cucurbit[6]uril (labelled CB[6] in the following) and the β-cyclodextrin (ascribed as β-CD). These are toroidal polymers as shown in schemes and molecular drawing from Figure 13 and Figure 14, with relatively large central holes. The computational approach of non-covalent association complexes meets two delicate problems: the long-range corrections [84], necessary in the use of DFT methods, and the so-called problem of Basis Set Superposition Errors (BSSE) [85,86]. An intrinsic drawback of the DFT is the wrong behavior of functionals at large distances from atomic nuclei [87,88], because of the empiric nature of the computational implementation. As a consequence, the pure DFT becomes unreliable at the estimation of van der Waals contribution [89,90,91]. A large variety of interventions are trying to alleviate this failure, from the attempt to redraw the functional in areas far from atoms [68,92,93], to the straight add of incremental terms, over the DFT direct results, adjusting the total energy (like in Grimme D2 and D3 versions [94,95,96,97]). Here, we will not perform tests regarding the various long-range corrections, trusting ourselves in Grimme type II or III post-computational adds, in all types of calculations. 

Based on chemical intuition and simple measurements of cavity size, one might assume that the formation of an encapsulation complex TEMPO@CB[6] is not convenient [93]. Indeed, the calculations are confirming a positive amount at the complex formation. Despite the non-bonded status, the complex can be optimized as relative minimum because of energy barriers that are faced at the displacement from the enforced configuration, because of tighter margins of the cucurbituril barrel.

Conversely, the energy balance is favourable to the formation of TEMPO@β-CD association, in line with the qualitative estimation of a larger inner room available at this host and according to experimental data [1,99]. In the evaluation of non-covalent associations, the computational practice invokes the so-called *counterpoise* treatments [85,86], which aim to correct the alleged BSSE problems. This means that, instead of taking as *E_A_* and *E_B_* the regular molecular calculations on monomers, must consider the E_A*_ and E_B*_ values resulted for rather artificial objects: molecule *A* in the presence of basis set of *B* (no electrons or nuclei from *B* side) and *vice versa*. Table 1 shows the Gaussian-type results for the ∆*E = E_AB_*− *E_A_* − *E_B_* and BSSE-corrected ∆*E_corr_ = E_AB_* − E_A*_ − E_B*_ energy differences, taken with various bases and the PBE (Perdew-Burke-Ernzerhof) functional.

Let us first discuss first the *AB* = TEMPO@CB[6] complex, optimized with the PBE functional and the 6-31+G basis, including Grimme D2 long-range corrections. The *A* and *B* are the TEMPO and cucurbit[6]uril units, taken at the same geometry like in the complex. One notes that the BSSE correction worsens the energy of the complex, adding positive shifts in the range 9–20 kcal/mol. The brute complexation energies are depending rather strongly on the basis set, varying between the 2.64 and 13.75 extrema (in kcal/mol). The variation is attenuated after the BSSE corrections, contained in the interval of 21.53 kcal/mol and 24.55 kcal/mol. While looking at the brute quantities, one may assume, for instance, when comparing the 6-31+G and 6-31G* with 6-31G, that richer bases lead to more positive formation energies. However, the trend does not hold, comparing e.g., the 6-31+G and 6-31++G couple. The same fact is seen when comparing 6-31G* and 6-31G**. The combined add of diffuse and polarization shells, like in 6-31+G* seems to have synergetic consequences, leading to shifts larger than the sum of those recorded in the 6-31+G and 6-31G* cases. In several instances, the *counterpoise*-type formation energies show a reverted trend than the brute ones, when basis is changed. Without the Grimme D2 correction, or with the D3 version, the systems result more non-bonded.

The system TEMPO@β-CD appears clearly bonded in all of the calculations, with formation energies estimated in the −29 to −37 kcal/mol range in the brute form and in the narrower −24 to −25 kcal/mol domain after the *counterpoise* correction. Without the D2 terms, the BSSE corrected formation energies of the TEMPO@β-CD are all slightly positive, ranging in the 2.1 to 4.14 kcal/mol domain. It appears then that the empiric long-range corrections are absolutely necessary for reaching the conclusion of favourable TEMPO-cyclodextrin complexation, as suggested by chemical intuition and experimental data [1,99]. Like in the TEMPO@CB[6] case, the variations of TEMPO@β-CD formation energies also cannot be clearly rationalized as function of type and amount of diffuse or polarization terms accessorizing the trunks of the 6-31G and 6-311G bases.

Facing the discussed synopsis, one might complain that the GTO bases have the disadvantage of non-systematic trends, because, in general, their intrinsic build-up is not conceived as a stepwise hierarchy. Other bases, for which a systematic design can be claimed, such as the series of cc-pv[*n*]z (with *n* = 2, 3 ... 6) are too demanding, unaffordable for large molecules.

In the situation of dilemmatic dependence on GTO basis sets, we propose, as a valuable alternative, the use of plane-wave (PW) methods. The PW DFT calculations are mostly used by physicists, in solid state problems and band electronic structure [100]. However, in the so-called Gamma point option, the methods can be employed for molecular systems, provided that these are neutral. Actually, in the computational engine, the system is treated as an infinite collection of boxes hosting the molecule, but if the size of the unit cell is sufficiently large, then the inter-cell effects are kept negligible.

For the sake of methodological considerations, we performed PW calculations with the ABINIT code [101,102,103] while using the PBE functional. For the TEMPO@CB[6] system, a cubic box with the *a* = 20 Å edge was considered to be sufficiently large, ensuring a minimal separation of about 7.8 Å between the closer atoms from neighbouring cells. The advantage of a PW calculation is the possibility of a fine and systematic tuning of the basis set quality. More concretely, the bases are defined by the maximal kinetic energy of the associated plane waves. As well known, the higher the energy, the smaller the wavelength, a fact related with better resolution in describing space-related quantities, such as the local variation of the electron density. The threshold related with basis quality can be continuously tuned by energy cut-off keywords (*e_cut_*). A systematic search along several *e_cut_* values can provide an extrapolation to results independent on the basis choice. Although the chemists are not very familiar with PW methods, we point with the occasion of selected example the advantage of the mentioned leverage.

Table 2 and Table 3 show the results of the PW calculations at different *e_cut_* values, for the TEMPO@CB[6] and TEMPO@β-CD, respectively. The results correspond to the geometry of the complex reoptimized in the PBE/PW setting, including the Grimme D2 long-range contributions. The new geometries are not differing much from the GTO results. One may note the monotonous trend in the *E_A_*, *E_B_*, *E_AB_*, and ∆*E* quantities, as a function of the cut-off energy. Showing a smooth decay, each curve (the component energies and their differences) can be fitted with an *E* = *E*_0_ + *a*⋅exp(−*b*⋅*e_cut_*) function. Subsequently, *E*_0_ is the extrapolated value of energy at infinite precision in the basis (*e_cut_* → ∞).

In the PW calculation, there is no need for a BSSE corrections because, as function of the *e_cut_* tuning parameter, the molecular box is filled with the same set of basis primitives for all the systems, *A* or *B* monomers and the *AB* complex. Subsequently, this treatment also acts as a critical assessment of the BSSE shifts, in the semi-quantitative comparison with GTO results. The PW and GTO calculations are not completely comparable, because of intimate differences in the nature of the basis, but one might say that the ∆*E*_∞_ = 20.27 kcal/mol value for the TEMPO@CB[6] complex, picked from the right-bottom corner of Table 2, is in the same range with the ∆*E_corr_* values found in Table 1. In spite of the alleged non-systematic variation, one may say that richer GTOs lead to ∆*E_corr_* approaching the 21 kcal/mol margin. Thus, keeping the same functional, PBE, and the same long-range terms, Grimme D2, the GTO and PW combined treatments, point to a positive complexation energy in the range of 20–21 kcal/mol, as an acceptable final estimation for TEMPO@CB[6].

Tuning the *e_cut_* parameter in PW calculations of the TEMPO@β-CD complex (see Table 3), the extrapolated energy of formation is −21.9 kcal/mol, in a range comparable with the BSSE estimation in the frame of GTO based calculations. This result is obtained with the Grimme D2 correction included. Without this, all of the formation values are shifted by 26.76 kcal/mol, ending then with a positive balance, like in the case of *counterpoise*-corrected Gaussian-type calculations. One finds again that the results are roughly comparable with those of BSSE GTO-based calculations in the proposed PW energy extrapolation procedures.

At the end of the section dedicated to complexation energies in model systems, let us consider the results obtained within the ADF code. The red line of the discussion compares the performance with different types of bases, GTOs vs. PW, adding now the STO case. We confine ourselves to the sole use of TZP basis and PBE functional, adding afterwards the Grimme D3 corrections (the D2 version is not available in ADF). The formation energy of TEMPO@CB[6] in the STO:TZP/PBE/D3 setting is positive, 36.64 kcal/mol. The GTO and STO worlds are not clearly comparable. Nominally, the TZP abbreviation, i.e., triple-zeta and polarization would correspond to the 6-311G* case, but this parallelism should be taken with caution.

The formation energy in a GTO: 6-311G*/PBE/D3 calculation is roughly comparable, 41.63 kcal/mol. The D3-type dispersion energies are very similar in the STO vs. GTO compared cases: −45.68 kcal/mol vs. −45.71 kcal/mol, given the resemblance of the optimized geometries. The certain differences in the discussed calculations appear then because of intimate details in the STO vs. GTO- based computational technologies. The BSSE correction in the TZP/PBE calculation brings an increment of 5.82 kcal/mol to the formation energy, which is definitely smaller than the income from *counterpoise* correction within the GTO series.

The ADF offers the advantage of energy decomposition analysis [104,105]. Thus, for TEMPO@CB[6], one finds that the electrostatic contribution seems favourable to complexation, leading to the −97.16 kcal/mol stabilizing amount. The orbital interactions, cumulating the weak and partial bonds established in long-range regime, are bringing −44.32 kcal/mol to the energy balance. This is accidentally close to the part due to the dispersion energy, mentioned before. All of these bonding contributions are superseded by the part due to the term, called Pauli repulsion, amounting +223.8 kcal/mol, which leads to the resultant unfavourable association energy. The Pauli repulsion is a quantum effect [106,107,108,109], expressing the mutual repeal of atomic cores, besides of what is expected from nuclear-nuclear or electron-electron classical Coulomb effects. It is ultimately due to the antisymmetric nature of multi-electron wave-functions, determined by the exclusion principle acting between fermions.

The ADF calculation of TEMPO@β-CD in a similar setting yields a net stabilization of −24.1 kcal/mol with the Grimme D3 corrections and a slightly non-bonded status, with 2.29 kcal/mol, without this ingredient. In this respect, the STO-based calculation shows similar trends with the GTO and PW procedures. The BSSE correction makes the formation energy more positive by 1.83 kcal/mol, which is smaller than the GTO equivalent.

In TEMPO@β-CD, all of the energy components show smaller absolute values than the TEMPO@CB[6] equivalent. The Pauli repulsion amounts only 35.9 kcal/mol. The electrostatic and orbital interactions are, respectively, −21.46 kcal/mol and −14.0 kcal/mol. Altogether, the nonbonding and bonding contributions are giving an almost null game, the cohesion being decided by the empirical dispersion term, −26.4 kcal/mol in this case.

When comparing the association energies extrapolated at infinite basis with different GTO-based estimations, one may note that the modest Gaussian choices perform satisfactorily. This provides the hint of their salient use instead of large GTO bases, saving, in this way, the computer effort.

We end at this point our excurse in computational experiments on inclusion complexes. A full discussion deserves the debate upon the role of the solvent, comparing again the SCRF shortcuts with more realistic box containing physical bodies of solvent molecules. As this is a demanding enterprise, we limit our contribution to the above academic models, retaining as interesting clue the newly proposed procedure of extrapolating formation energies from PW calculations with progressively increased basis cut-off threshold.

## 3. Methods

Throughout this work, we employed several methods of electronic structure calculations in the frame of Density Functional Theory, under various setting and different codes, each choice being specified in the respective sections. A part of the calculations were done with the Gaussian 09 program [69], complemented with Orca suite [75], choosing as basis, from case to case, either Gaussian type orbitals (GTOs) from Pople’s class (e.g., 6-31+G or 6-311+G*) [83] or the EPR-II and EPR-III bases [78], adjusted for the estimation of hyperfine coupling parameters.

For the sake of methodological considerations, methods that are based on Slater type orbitals (STOs) were used, on the platform of ADF code (Amsterdam Density Functional) [80,81]. We also employed Plane Wave (PW) methods, with norm-conserving pseudo-potentials, using the Abinit code [101,102,103].

When the numeric experiments were constructed in the frame of GTO only calculation, we often relied on the popular B3LYP hybrid functional. When comparisons with ADF or PW results were concerned, we turned to Gradient corrected functionals or PBE [110], avoiding the use of hybrid methods, although the new versions of these codes are also including such options. The long-range corrections were introduced via CAM-B3LYP [68] or by Grimme’s D2 and D3 increments [94,95,96,97].

## 4. Conclusions

In this work, we propose a walk around topics regarding the state of the art computational approach of the quantities met in the practice of EPR spectroscopy of nitroxide-based radicals. Thus, we debate the calculation of exchange coupling parameters *J*, the Zero Field Splitting emerging from dipolar effects and the hyperfine electron-nucleus interaction. We also touch the problem of host-guest complexes formed between toroidal prototypic molecules and radicals.

The discussion tries to assess and prospect how the actual ab initio methods, working in molecular regime, can be used to pass the border toward the supramolecular systems and nano-scale assemblies. The proposed case studies are yet simplified structures, at the very margin of the nanoscale and accounting in a limited way the complications met in a real system, for instance kinetics and thermodynamics intervening for radicals in solution or other extended associations. However, we hope that the detective story that is constructed here is a valuable step toward the desiderata of magneto-structural correlations in dynamic systems and in the soft chemistry.

Although a relatively simple deal, nobody treated yet the explicit modeling of the exchange coupling between nitroxide spin carriers as function of their distance and angular mutual placement. We draw such correlations for a pair of TEMPO radicals, taken in different mutual configurations. The very interesting surprise is that the pattern of the magneto-structural relationships is qualitatively retrieved in the cases where a dichloromethane molecule is placed in between, when checking the role of the solvent in mediating the long-range coupling.

As function of its orientation towards the radical centres, the solvent can magnify, with several orders, the strength of the coupling, retaining its sign (i.e., conserving the ferro or antiferro situation with respect of pristine TEMPO dimers), or transmuting the nature of the exchange, e.g., from ferro to antiferro. Such conclusions should be very specific with respect of considered solvent, here confining only to the CH_2_Cl_2_ example. An extended mapping of features of different solvents would be an interesting challenge.

The solvent intervention also changes the dipolar coupling and ZFS parameters that are expected in solution, in comparison with those computed in the vacuum molecular models, but with a lesser extent, keeping all of the results in the same range of magnitude. At the same time, the ab initio ZFS parameters are resulting close to the estimation by simpler models, based on the variation of dipolar coupling with the third inverse power of separation between the spin carriers. Checking the role of solvent, we have seen that simplified solvent models from the SCRF class, implemented in the actual quantum chemistry codes, are of no use in the salient estimation of exchange or dipolar coupling.

We paid a special attention to the computational fundaments of the hyperfine coupling. The nowadays quantum chemistry acknowledges the subtle details of this problem designing special basis sets (for instance, EPR-II and EPR-III in the Gaussian-type methods). A good basis dedicated to this goal should be accurate both in the area close to the nucleus and in its long-range tail, while most of the current options are well tailored only for the valence radial part. We illustrated this issue proposing, as seminal example, a new Slater-type basis for the nitrogen atom (the site where the hyperfine coupling is manifesting in nitroxide) with properly designed behavior in all of the radial domains, particularly with reasonable amplitudes in the long-range outskirts.

We added methodological considerations to the analysis of host-guest formations, because this problem is found in the experimental use of EPR in spin-probe measurements. We dealt with the question of computed complexation energy, choosing two ring-shaped molecules as guests. The cucurbit[6]uril, (CB[6]) served as example for a too small cavity, where the formation of the complex has positive enthalpy, while β-cyclodextrin (β-CD) was taken as the situation where the encapsulation is convenient, as energy balance. Because the use of GTO establishment proves to be non-systematic as function of basis set, also demanding a somewhat questionable procedure, in the so-called *counterpoise* calculation of the formation energy, we proposed a different route to this quantity. We exemplified the use of Plane-Wave based calculation of complexes and of their molecular components, varying the threshold of an intrinsic parameter of the calculation, the kinetic energy cut-off. The energy results, as function of this cut-off, are varying in a very clear exponential pattern that allows for an extrapolation to what would represent molecular energies computed at an infinitely good quality of the basis set. In this way, one may obtain the formation energy at a conceptual set-up better than the actual *counterpoise* scaffolding.

An honest stand should acknowledge that the full quantum computation cannot be yet practiced at very large size, in the aperiodic, amorphous, or dynamic structural conditions met in the soft or nano domains of physics and chemistry. The existing approximate methods aim to attain the chemical realism in modeling, such as solvent models or molecular mechanics, are not satisfactorily acting in particular domains, like the rationalization of EPR in complex systems. However, clues that are suggested in the presented numerical experiments are offering hopes and incentives that a new generation of models for nanoscale is possible, if succeed to pass the molecular-supramolecular border in a legitimate way.

## Figures and Tables

**Table 1:** Brute (∆*E*) and BSSE-corrected (∆*E_corr_*) complexation energies of the TEMPO@CB[6] and TEMPO@β-CD embedded systems, computed with the PBE functional and Grimme D2 long-range increments. The values without the D2 treatment are obtained adding the +64.51 kcal/mol shift to all ∆*E* and ∆*E_corr_* values of the CB[6] adduct and +27.85 kcal/mol for the β -CD series. The situation of Grimme D3 corresponds to the +18.8 kcal/mol, respectively +1.46 kcal/mol shift of all the presented values.

Basis/AB	TEMPO@CB[6]		TEMPO@ β -CD	
	∆E (kcal/mol)	∆E_corr_ (kcal/mol)	∆E (kcal/mol)	∆E_corr_ (kcal/mol)
6-31G	3.91	24.55	−36.51	−25.09
6-31+G	6.33	23.61	−34.98	−25.75
6-31++G	6.06	23.62	−34.72	−25.73
6-31G*	4.15	24.26	−34.75	−23.71
6-31+G*	13.75	22.76	−29.39	−24.52
6-31++G*	13.65	22.77	−29.45	−24.51
6-31G**	2.64	22.99	−35.12	−24.33
6-31+G**	12.63	21.53	−29.53	−25.17
6-31++G**	12.56	21.53	−29.53	−25.15
6-311G	4.73	23.73	−33.60	−25.00
6-311+G	4.76	24.34	−34.91	−25.24
6-311++G	4.86	24.46	−35.17	−25.23
6-311G*	5.58	22.83	−32.30	−24.19
6-311+G*	13.09	23.19	−30.16	−24.54
6-311++G*	13.21	23.25	−30.29	−24.52
6-311G**	5.14	22.07	−32.68	−24.91
6-311+G**	13.1	22.1	−29.70	−25.23
6-311++G**	13.17	22.13	−29.64	−25.22

**Table 2:** Molecular and complexation energies computed with plane-waves for the *AB*=TEMPO@CB[6] system, as function of the *e_cut_* threshold, parallel with the accuracy of the basis set. The data correspond to the complex optimized with PBE functional and Grimme D2 corrections. The values without the D2 treatment are obtained adding the +63.17 kcal/mol shift to all ∆*E* values.

*e_cut_* (a.u.)	*E_A_* (a.u.)	*E_B_* (a.u.)	*E_AB_* (a.u.)	∆*E* (kcal/mol)
10	−89.752721	−666.521144	−756.272743	0.704
15	−91.420119	−682.677029	−774.078638	11.615
20	−91.955313	−688.117167	−780.046476	16.318
25	−92.127097	−689.949014	−782.046661	18.480
30	−92.182236	−690.565848	−782.717044	19.477
…	…	…	…	…
∞	−92.208300	−690.879000	−783.055000	20.269

**Table 3:** Molecular and complexation energies computed with plane-waves for the *AB*=TEMPO@β-CD system, as a function of the *e_cut_* threshold. The data correspond to the complex optimized with PBE functional and Grimme D2 corrections. The values without the D2 treatment are obtained adding the +26.76 kcal/mol shift to all ∆*E* values.

*e_cut_* (a.u.)	*E_A_* (a.u.)	*E_B_* (a.u.)	*E_AB_* (a.u.)	∆*E* (kcal/mol)
10	−89.808568	−843.193164	−933.080225	−49.255
15	−91.429465	−864.977950	−956.457872	−31.662
20	−92.003204	−874.457998	−966.508544	−29.707
25	−92.155612	−877.393910	−969.588669	−24.565
30	−92.187830	−878.113239	−970.339341	−24.016
35	−92.193473	−878.238044	−970.469690	−23.954
40	−92.195163	−878.253100	−970.486430	−23.950
…	…	…	…	…
∞	−92.213100	−878.724000	−970.972000	−21.900
